# Water Water Everywhere

**DOI:** 10.1100/tsw.2000.14

**Published:** 2000-12-15

**Authors:** Jean Levy

## Abstract

During the last decade of the 20th century the world was exposed to increasing episodes of extreme weather. Figures reveal a 0.6°C rise in average temperatures since records began in 1860, with the 1990s being the warmest decade and 1998 the warmest year. Experts believe that these rising temperatures, or global warming, are in part due to human influences.

Constant heat energy from the sun, in the form of short-wave radiation, reaches the surface of the Earth, but much of it is emitted back into space as long-wave radiation. Warming occurs when radiation does not escape, but is instead held in the atmosphere by gases such as water vapour, carbon dioxide, nitrous oxide, methane and other industrially produced substances. These are the so-called greenhouse gases; the retention of heat energy is called the greenhouse effect. Carbon dioxide is the most significant of the greenhouse gases. Its average concentration in the atmosphere has increased dramatically over the last 2 centuries in association with man's industrial and farming activities and land usage. Figures put this increase at around 30%. The corresponding warming of the atmosphere warms the oceans and ice sheets. The seawater undergoes thermal expansion, causing a rise in sea levels, made worse by the melting ice. As the ice sheets become smaller they are able to reflect less light, causing even greater temperature increases. Some scientists predict that all Arctic ice will have disappeared by late this century. There are also fears that the melting Arctic tundra will release trapped organic matter that, upon oxidation, will produce further quantities of greenhouse gases.

Certain factors have kept carbon dioxide concentrations in check. Plants continue to remove carbon dioxide from the air during photosynthesis, and replace it with oxygen. However, increasing production of carbon dioxide, due to the burning of fossil fuels and intensive farming practices, is increasingly outstripping its removal by plants. To make matters worse deforestation, urbanization and desertification are constantly diminishing the mass of plants able to photosynthesize. Some experts fear a 'runaway greenhouse effect' in which the factors causing global warming finally swamp the factors that prevent it. It has been suggested that vast forest planting schemes should be undertaken, but scientists are not certain how successful this would be. They warn that the biosphere cannot suddenly deal with all the carbon released from ancient deposits of coal, oil and gas. They further warn that to plant trees with dark leaves and bark, in otherwise snowy or bright areas (such as Siberia), would effectively darken the Earth's surface causing more heat to be absorbed, thus contributing to global warming. In addition, plants themselves emit carbon dioxide due to respiration; during periods of darkness, plants would cause an overall increase in the carbon dioxide load.

This carbon dioxide theory of climate change has been known for decades. For many years policy makers preferred to interpret the warnings as the ramblings of environmentalist cranks. However, over the last 20 years, the miserable possibility that they might have been half correct has dawned. Fears filtered upwards as extreme temperatures, increased precipitation, drought and frequent hurricanes provided records that were too soon broken. International policy makers began to talk about talking.

In 1992, at the Earth Summit in Rio de Janeiro, the United Nations Framework Convention on Climate Change (UNFCCC) was opened for signature. It offered a global strategy on climate change, detailing concerns about the melting of the polar ice caps and rising sea levels. In 1997 the Kyoto Protocol was established, promoting international cooperation towards more climate-friendly policies and technologies. It set up targets and timetables for a worldwide reduction of carbon-based gas emissions by an average of 5.2% below 1990 levels during the 5-year period 2008-2012, with various nations adopting different targets: 6% for Japan, 7% for the US and 8% for the EU. Other nations, such as Russia and the Ukraine, agreed to stabilize at 1990 levels. Environmentalists demanded that these reductions were inadequate and that cuts of 60-80% were required - and required immediately. For the Protocol to have effect, it had to be ratified by 55 parties to the Climate Change Convention, including at least some industrialized countries.

From November 13-23 2000, representatives of 180 countries gathered in The Hague for a 2-week international meeting on climate change, i.e., the Sixth Session of the Conference of the Parties to the Convention, or COP6. Its aim was to encourage the various countries to accept the terms of the Kyoto Protocol and so commit to combat climate change. By the start of the conference, most governments had not ratified the Protocol, preferring to wait until the conference for agreement on how the Protocol could work in practice. The Hague conference was seen as a make or break opportunity for global action on climate change. It was hoped that the conference would tie up loose ends, that developing country delegations would be reassured of support for their efforts, and that all parties would subsequently launch or

Previously posted in NewsLab, TheScientific World.
© 2000 with author.

continue with processes leading to reduced emissions. Political agreements were needed. The conference, which had always promised to be politically difficult and technically complex, failed to find any agreement and, in line with environmentalist predictions, finally collapsed. The EU and the US remained at loggerheads. Analysis of The Hague debate is a journey into the absurd. It would be truly humorous if the situation were not so desperate. Whilst the climate seemed to be undergoing noticeable changes for the worst in parts of Europe, conference delegates fought to argue their way out of previous commitments, seemingly more obsessed with the minutiae of environmental semantics than the environmen

Without the cooperation of the US, which accounts for around a quarter of the world's total output of carbon dioxide, there was never much likelihood of success. Most developed countries were concerned that a transition to a low-emission economy would damage competitiveness. The US Senate had previously passed a resolution that US ratification of the Kyoto Protocol would be conditional upon assurances that US competitiveness would not be harmed. In this respect, the US was backed by Canada, Australia, New Zealand and Japan (the so-called Umbrella Group), as well as oil companies, China, and the Opec nations, who feared reduced oil exports. The countries of the Umbrella Group came to the conference with schemes that would spare them from actually reducing industrial carbon emissions. Instead they called for new forests and crops at home, and the aiding of forestation projects abroad, to be counted as carbon credits. The possibility of using trees and crops as 'carbon sinks' was outlined in the Kyoto Protocol. The US, Canada and Japan suggested 'additional activities' to avoid reducing actual emissions, including urban greening, and the development of crops specifically engineered to soak up carbon dioxide. However, the idea of carbon sinks did not go down well with the EU, many of the developing countries (G77), and most non-governmental agencies. They argued that planting trees alone could not save the global climate, and called for real domestic cuts in emissions. The situation worsened when it became obvious that the US was seeking not only to plant new forests to gain 'carbon credits', but also to be allowed to gain credits for the amount of carbon soaked up by existing forests and farmlands. The EU immediately rejected this proposal. The US compromised by suggesting that all states should be allowed to count, as credits, some of the carbon dioxide their existing forests absorb. The EU rejected the compromise, arguing that if the US proposals were to be adopted, they would allow for an 8-9% increase over 1990 emissions. The Swedish Environment Minister, pointed out that Sweden and Finland, Europe's most forested countries, would, under such a scheme, be able to increase their emissions by 30-40%. Japan and Canada added to the general feeling of circus by calling for rules that would allow them to avoid counting increased emissions from cutting down forests, but to count the carbon credits if the deforested areas were replanted. At best this would encourage the destruction of established mixed forests and their replacement by monoculture plantations.

A number of other flexible mechanisms lurking within the Kyoto agreement allowed industrialized countries to avoid domestic action by acquiring emission credits abroad. Joint Implementation would allow projects between developed countries, whereby one country would contribute to the reduction of emissions in another, and so gain emissions credits. This included assistance with the development of nuclear power plants instead of power plants using fossil fuels, and was therefore unpopular with environmentalists. A Clean Development Mechanism would also allow for similar projects between developed and developing countries, enabling the developed country to claim emission credits.

The US called for the conference to accept Carbon Trading mechanisms, also outlined in the Protocol. International Emissions Trading would allow developed countries exceeding their emissions reductions target to sell their surplus carbon credits to countries unable to meet their commitments. In this way richer, developed countries could avoid domestic reductions each year by buying tonnes of carbon credits from countries like Russia, who produce less carbon dioxide than their Kyoto targets, due to the collapse of their economies. The EU was not happy with carbon trading.

By the end of the conference there had been no agreement; the delegates went home, making way for the conference booked for the following week. Just in case there had been an agreement, Japan and Russia had opposed binding commitments. It was suggested that countries that failed to fulfil their obligations would able to deduct excess emissions from the next period budget, without further sanctions. There would have been no guarantees anyway.

So, where to now? It is really up to the US. The American public has a very high level of environmental awareness, and states such as California have taken pioneering environmental initiatives. But Washington, D.C. is guided by the interests of industry and the oil companies. President-elect Bush will probably recommend withdrawal from the Kyoto agreement. It is rumoured that he does not believe in global warming anyway. But then neither do some of the world's largest companies including Exxon (which does not recognize climate change), Texaco and DuPont. This is of course all for the best, because if these guys did believe in global warming, they would have to feel very bad about their behaviour over the last few years. The climate change talks resume in Bonn in May 2001, when all will be focused on persuading the US - if it is there - and its allies to alter their position. If the Americans do pull out of the fray there will be no point in carrying on.

Sadly, at the moment nothing is making the situation better. The shocking conclusion must be that no international group of politicians or policy makers has the situation under control. We have no global strategy to combat climate change and no superhero to 'save the planet'.

So what can WE do?

It seems that we have the following options: resignation, protest or prayer. The British are good at all these things. We are also good at weather. We get a lot of it being where we are. We have the Gulf Stream, the tail end of hurricanes and a continuum of weather fronts rippling across the Atlantic. We have blizzards, gales, droughts and fog - sometimes all in the same day. We discuss the weather, moan about the weather, celebrate the weather and have a wealth of customs and folklore that reveal our obsession with the weather. But we have had some very extreme weather here in the last few weeks and we are quite worn out by it. In November the ancient Roman city of York was barely saved from the worst floods and storms since 1625. The climate over the UK has definitely altered and we have all noticed it. We have had ferocious storms, devastating winds, torrential rains and floods, the like of which no one can remember. Is this all part of the package that the Doomsday ecologists predicted?

A recent bout of extreme weather hit the UK on Sunday, November 5th. People were warned to stay inside as the weather front, casually referred to as Armageddon, hit the UK. It was Guy Fawkes night, and as stalwart British patriots were attempting to light bonfires and burn effigies of the man who tried to blow up the Houses of Parliament, I drove up the valley through the torrential rain and flash floods to the Chilgove Bonfire party. The local kids were throwing diesel onto a giant pile of drenched wood and hay in an attempt to set it alight. The local elders were huddled in the White Horse Inn, debating the possibility of lighting aerial fireworks in gale-force winds. We decided against it. The kids did manage to get the bonfire started and we watched the flames leap up and turn to steam as the dewpond outside turned into a lake and waves lapped over the burning hay.

By the following day the gales had decreased, but the trouble had just begun. That amount of water, falling on the Chalky South Downs, had nowhere to go other than downhill. The rain stopped but the water table rose and kept on rising until it was 5 meters above normal and therefore above ground level. This meant that the valley was officially under water. Fresh water springs burst up through the floor in people's houses, through the surface of the road causing potholes and through the cellar of the White Horse Inn. Pumping simply recycled the water out of the bar into the dewpond and back into the cellar. The road leading to Chilgrove became the bed of a torrential river, all 3 miles of it. For a couple of days it was still possible to drive through it, although it took half an hour to do so. That gives you a lot of time to think - particularly about water.

This was not a river bursting its banks, or even rising sea levels. This was so much water that it was taller than the land. That throws a whole new light on the torrential rain aspects of global warming. What can you do with that much water? The mind strays into the realms of the surreal. What if every man, woman and child each owned 3 buckets of water - would that help? What if the people fortunate enough to be on mains drainage flushed their buckets down the toilet? What if those people discussing climate change actually did something to stop it? Now that's really surreal.

The water levels have now started to fall, and the roads have re-opened, although experts are warning that we've seen nothing yet. People are thinking of other things, as they do. Getting on with their lives assuming that superior minds somewhere will save the day. I have heard that US agricultural scientists are investigating the possibility of feeding sheep, pigs and cows special anti-flatulence diets to reduce levels of methane. Now that makes my 3-bucket plan sound really credible.

Jean Levy
